# Global Phylogeography and Genomic Epidemiology of Carbapenem-Resistant *bla*_OXA-232_–Carrying *Klebsiella pneumoniae* Sequence Type 15 Lineage

**DOI:** 10.3201/eid2911.230463

**Published:** 2023-11

**Authors:** Yuye Wu, Tian Jiang, Xianhong He, Jiayu Shao, Chenghao Wu, Weifang Mao, Huiqiong Jia, Fang He, Yingying Kong, Jianyong Wu, Qingyang Sun, Long Sun, Mohamed S. Draz, Xinyou Xie, Jun Zhang, Zhi Ruan

**Affiliations:** Zhejiang University School of Medicine Sir Run Run Shaw Hospital, Hangzhou, China (Y. Wu, T. Jiang, X. He, J. Shao, C. Wu, W. Mao, Y. Kong, X. Xie, J. Zhang, Z. Ruan);; Wenzhou Medical University Affiliated Wenling Hospital, Taizhou, China (T. Jiang);; Third People's Hospital of Xiaoshan District, Hangzhou (X. He, J. Shao);; Shaoxing University Affiliated Hospital, Shaoxing, China (W. Mao);; Zhejiang University School of Medicine First Affiliated Hospital, Hangzhou (H. Jia);; Key Laboratory of Clinical In Vitro Diagnostic Techniques of Zhejiang Province, Hangzhou (H. Jia);; Zhejiang Provincial People's Hospital, Hangzhou (F. He);; Key Laboratory of Precision Medicine in Diagnosis and Monitoring Research of Zhejiang Province, Hangzhou (Y. Kong, X. Xie, J. Zhang, Z. Ruan);; Zhejiang University School of Medicine Fourth Affiliated Hospital, Hangzhou (J. Wu);; No. 903 Hospital of PLA Joint Logistic Support Force, Hangzhou (Q. Sun);; Hangzhou Women's Hospital, Hangzhou (L. Sun);; Case Western Reserve University School of Medicine, Cleveland, Ohio, USA (M.S. Draz);; Cleveland Clinic, Cleveland (M.S. Draz)

**Keywords:** Klebsiella pneumoniae, antimicrobial resistance, carbapenem resistance, Enterobacterales, carbapenemases, *bla*_OXA-232_, bacteria, healthcare-associated infections, pneumonia, bloodstream infections, wound site infections, meningitis, respiratory infections, genomic epidemiology, phylogenetic, phylodynamic, China

## Abstract

Prevalence of carbapenem-resistant *Klebsiella pneumoniae* (CRKP) has compromised antimicrobial efficacy against severe infections worldwide. To monitor global spread, we conducted a comprehensive genomic epidemiologic study comparing sequences from 21 *bla*_OXA-232_–carrying CRKP isolates from China with *K. pneumoniae* sequence type (ST) 15 strains from 68 countries available in GenBank. Phylogenetic and phylogeographic analyses revealed all *bla*_OXA-232_–carrying CRKP isolates belonged to ST15 lineage and exhibited multidrug resistance. Analysis grouped 330 global *bla*_OXA-232_–carrying ST15 CRKP strains into 5 clades, indicating clonal transmission with small genetic distances among multiple strains. The lineage originated in the United States, then spread to Europe, Asia, Oceania, and Africa. Most recent common ancestor was traced back to 2000; mutations averaged ≈1.7 per year per genome. Our research helps identify key forces driving global spread of *bla*_OXA-232_–carrying CRKP ST15 lineage and emphasizes the importance of ongoing surveillance of epidemic CRKP.

The global spread of carbapenem-resistant Enterobacterales, particularly carbapenem-resistant *Klebsiella pneumoniae* (CRKP), poses a severe and ongoing public health threat because of high rates of illness and death (*1*). The production of carbapenemases is the most crucial carbapenem-resistance mechanism in gram-negative bacteria worldwide (*2*). One of the most prevalent carbapenemase genes, *bla*_OXA-48_ (oxacillin-hydrolyzing β-lactamase), is found predominantly on large and transferable plasmids in bacteria of order Enterobacterales. Prevalence of *K. pneumoniae* strains carrying a *bla*_OXA-48_–like gene in China increased during 2018–2022 (*3**–**6*). Nosocomial outbreaks, including among pediatric patients, have called attention to the importance of better understanding the transmission potential of CRKP isolates carrying the *bla*_OXA-48_–like gene (*4*–*6*). 

*bla*_OXA-232_, a variant among *bla*_OXA-48_ genes with limited carbapenem hydrolytic activity, was initially identified in France in 2011 (*7*). Most *bla*_OXA-232_ genes have been discovered on small and nonconjugative ColKP3-type plasmids (*8*). In China, some strains of sequence type (ST) 15, the predominant lineage of *bla*_OXA-232_–carrying *K. pneumoniae*, mediate low-level carbapenem resistance and many coexist with virulence plasmids (*6*,*9*,*10*). Applying a previously proposed reverse genomic epidemiology strategy enabled us to make genome comparisons of isolates to identify shared sources of infection on the basis of genomic similarity (*11*). This approach, developed in response to increasing rates of global spread of antimicrobial-resistant bacteria, emphasizes the need to explore transmission of infection from a global rather than a country- or case-specific perspective. Limited data from previous studies on the number and geographic diversity of *bla*_OXA-232_–carrying *K. pneumoniae* isolates has impeded full understanding of the genomic evolution and transmission dynamics of CRKP (*5*,*12*). 

We aimed to perform a multicenter molecular epidemiologic study of carbapenem-resistant *bla*_OXA-232_–carrying *K. pneumoniae* ST15 isolates in China, focusing on the genomic characterization of the lineage and the genetic context of the *bla*_OXA-232_–carrying plasmids. We performed large-scale comprehensive genomic epidemiologic analysis to investigate the transmission histories, common ancestors, evolution rates, and population structures of all publicly available *bla*_OXA-232_–carrying *K. pneumoniae* of ST15 lineage. Our study provides data that will help monitor global spread and the epidemic expansion of *K. pneumoniae* ST15 lineage and provide insights for developing new infection control strategies. The ethics committee of Sir Run Run Shaw Hospital, Zhejiang University School of Medicine, approved this study (2022–0227). 

## Methods

### Isolate Collection

We performed a retrospective microbiologic characterization of 21 *bla*_OXA-232_–carrying CRKP isolates obtained from a collection of 2,398 nonduplicate *K. pneumoniae* clinical isolates recovered from 5 hospitals across 3 areas of Zhejiang Province, China, during August 2018–March 2022. Presence of the *bla*_OXA-232_ gene was detected by PCR and validated by Sanger sequencing (*13*). We identified bacteria using VITEK 2 (bioMérieux, https://www.biomerieux.com) and matrix-assisted laser desorption/ionization-time-of-flight (MALDI-TOF) mass spectrometry (Bruker, https://www.bruker.com). 

### Antimicrobial Susceptibility Testing 

We performed antimicrobial susceptibility testing (AST) using a broth microdilution method on antimicrobial agents amikacin, aztreonam, fosfomycin, cefoxitin, cefepime, cefotaxime, cefiderocol, levofloxacin, imipenem, meropenem, polymyxin, tigecycline, and ceftazidime/avibactam. We determined MICs for cefiderocol using iron-depleted cation-adjusted Mueller-Hinton broth in custom-prepared MIC panels (*14*). We interpreted antimicrobial susceptibility breakpoints according to Clinical and Laboratory Standards Institute 2020 (https://clsi.org) and EUCAST 10.0 (https://www.eucast.org/clinical_breakpoints) guidelines. We used *Escherichia coli* ATCC 25922 as a quality control strain for AST. 

### Plasmid Conjugation

We used rifampin-resistant *E. coli* EC600 as the recipient. We selected transconjugants on Mueller-Hinton agar supplemented with rifampin (300 mg/L) and imipenem (4 mg/L). We counted numbers of transconjugant colonies after overnight incubation at 37°C and confirmed transconjugants using PCR and Sanger sequencing. 

### Whole-Genome Sequencing

We performed whole-genome sequencing using the short-read Illumina HiSeq X10 (https://www.illumina.com) with 150-bp paired-end protocol. We also sequenced the representative isolate (KP3295) selected from the 21 CRKP isolates on the long-read Oxford Nanopore MinION platform (Nanopore Technologies, https://nanoporetech.com). We assembled the derived short Illumina reads and long MinION reads using Unicycler version 0.4.8 (https://github.com/rrwick/Unicycler) (*15*). 

### Genomic Features and Plasmid Characterization

We annotated genomes using the National Center for Biotechnology Information (NCBI) Prokaryotic Genomes Annotation Pipeline (https://github.com/ncbi/pgap). We determined antimicrobial resistance genes using ABRicate version 1.0.1 (https://github.com/tseemann/abricate) against the NCBI AMRFinder database (*16*) and plasmid replicon types using the PlasmidFinder database (*17*). We detected virulence determinants using Kleborate version 2.1.0 (*18*) and the 2022 Virulence Factor Database (*19*). We performed in silico multilocus sequence typing (MLST) analysis and bacterial source tracking using BacWGSTdb 2.0 (*20*). We used EasyFigure (*21*) and BLAST Ring Image Generator (*22*) to analyze genetic context and homologous regions of *bla*_OXA-232_ among the isolates. 

### Phylogenetic Analysis

On June 3, 2022, we retrieved genome sequences of 43,402 *K. pneumoniae* strains from 114 countries and related clinical metadata from the NCBI pathogen detection portal (Appendix 1). After conducting in silico MLST analysis and detecting the presence of *bla*_OXA-232_ gene in *K. pneumoniae*, we selected 330 *bla*_OXA-232_–carrying *K. pneumoniae* ST15 strains for further investigation. On the basis of geographic information for each isolate, we created a minimum-spanning tree based on core genome MLST allelic profiles of *K. pneumoniae* ST15 isolates using chewieSnake (*23*) and visualized the phylogenetic tree using GrapeTree (*24*). We considered isolates to be closely related genotypically when separated by a distance of ≤20 alleles. We used Snippy version 4.6.0 (https://github.com/tseemann/snippy) to align sequences and identify single-nucleotide polymorphisms (SNPs). We parsed genome alignment through Gubbins (https://github.com/nickjcroucher/gubbins), which identified and removed recombination regions, and inferred and constructed a maximum-likelihood phylogeny from those SNPs. We calculated pairwise SNP distances between the genomes of each isolate to define clades. 

### Phylodynamic Analysis 

We performed root-to-tip regression analysis using TempEst version 1.5.3 (https://github.com/beast-dev/Tempest) to confirm that the maximum-likelihood tree had sufficient temporal signal. We used a Bayesian Markov Chain Monte Carlo approach implemented in BEAST2 (*25*) to analyze alignment of putative substitution mutations identified by Gubbins, on which estimates the time-scaled phylogenetic tree was based, as well as time of most recent common ancestor (tMRCA) and mutation rates. We allowed each run 300 million iterations, sampled from every 20,000th iteration, and discarded the first 10% of the samples as burn-in. We estimated the effective sample size using Tracer version 1.7.1 (https://github.com/beast-dev/tracer/releases) to evaluate the operation convergence. We used RhierBAPS version 1.0.1 (*26*) to analyze the allele frequency parameters of the bacterial population and cluster individual sequences to identify the population structure, then used the resulting clades as input for the SkyGrowth package (https://github.com/mrc-ide/skygrowth) to evaluate effective population sizes. We visualized and annotated phylogenetic trees using the interactive Tree of Life (iTOL) V5 web server (*27*). We analyzed and visualized transmission links for isolates and nodes with spatial phylogenetic reconstruction of evolutionary dynamics using SpreaD3 (*28*) under a discrete trait model. We deposited genome sequences of the 21 CRKP isolates in GenBank (BioProject accession nos. PRJNA818898 and PRJNA745926). 

## Results

### Clinical and Microbiologic Characteristics

During August 2018–2022, we recovered *bla*_OXA-232_–carrying *K. pneumoniae* strains from 21 of 2,398 *K. pneumoniae* patients among 5 hospitals in Zhejiang Province, an incidence rate of 0.87% (Table 1). Most patients with diagnosed *K. pneumoniae* infections experienced pulmonary infections accompanied by respiratory failure. According to AST data (Table 2), most of the *bla*_OXA-232_–positive isolates were resistant to amikacin, aztreonam, fosfomycin, imipenem, meropenem, cefoxitin, cefepime, cefotaxime, and levofloxacin. Those isolates showed low-level resistance to carbapenems but remained susceptible to colistin, tigecycline, ceftazidime/avibactam, and cefiderocol. 

**Table 1:** Characteristics of 21 infected patients in China investigated in study of global phylogeography and genomic epidemiology of *bla*_OXA-232_–carrying carbapenem-resistant *Klebsiella pneumoniae* sequence type15

Isolate	Age, y/sex	Clinical diagnosis	Specimen source	Isolation date
KP3	78/M	Atrial fibrillation, severe pneumonia, respiratory failure	Urine	2020 Nov
KP41	66/M	Cardiac failure, respiratory failure, urinary tract infection	Urine	2020 Nov
KP105	61/F	Cholangiolithiasis	Bile	2020 Nov
KP232	86/M	Cholangiolithiasis	Urine	2020 Dec
KP306	42/M	Severe acute pancreatitis	Excreta	2020 Dec
KP5	61/M	Hydrocephalus	Urine	2021 Jan
KP441	76/F	Lung infection, heart failure	Sputum	2021 Jun
KP68	84/F	Chronic obstructive pulmonary disease	Bronchoalveolar lavage fluid	2021 Sep
KP76	47/F	Posttraumatic brain syndrome	Blood	2021 Sep
KP77	73/M	Multiple brain contusion and laceration	Sputum	2021 Sep
KP79	54/M	Chronic obstructive pulmonary disease	Sputum	2021 Sep
KP81	94/M	Cerebral infarction, hypertension	Sputum	2020 Aug
KP85	56/M	Inhalation lung injury	Sputum	2021 Oct
KP89	56/M	Inhalation lung injury, respiratory failure	Excreta	2021 Nov
KP91	68/M	Respiratory failure, diabetes	Urine	2020 Dec
KP97	55/F	Exogenous injury, motor neuron disease, respiratory failure	Sputum	2021 Dec
KP9112	86/F	Chronic obstructive pulmonary disease	Sputum	2020 Sep
KP135	76/M	Cervical spinal fractures, high paraplegia	Sputum	2021 Jan
KP8474	49/M	Intracranial space occupying lesions	Sputum	2020 Jul
KP12339	65/M	Intracranial injury	Sputum	2019 Jan
KP3295	52/M	Paraplegia	Urine	2018 Aug

**Table 2:** MICs for different antimicrobial drugs of 21 *bla*_OXA-232_–carrying sequence type 15 carbapenem-resistant *Klebsiella pneumoniae* isolates from patients in China *

Isolate	MIC, mg/L												
	AMK	ATM	FOF	FOX	FEP	CTX	LVX	IPM	MEM	CST	TGC	FDC	CZA
KP3	>128	>128	>128	64	>64	>128	64	8	4	<0.0625	1	2	0.25/0.125
KP41	>128	>128	>128	16	>64	>128	16	4	2	<0.0625	0.25	2	0.5/0.25
KP105	>128	>128	>128	128	>64	>128	128	>128	>128	<0.0625	2	1	0.5/0.25
KP232	>128	>128	128	128	>64	>128	>128	8	4	0.0625	2	0.5	0.5/0.25
KP306	>128	>128	>128	64	>64	>128	64	4	4	<0.0625	1	4	0.25/0.125
KP5	>128	>128	>128	64	64	>128	32	2	4	0.5	2	0.25	2/1
KP441	>128	>128	>128	32	>64	>128	16	0.5	2	0.125	2	1	2/1
KP68	>128	>128	>128	64	>64	>128	32	32	32	0.5	1	1	4/2
KP76	>128	>128	>128	32	>64	>128	16	1	1	0.25	1	1	1/0.5
KP77	>128	>128	>128	32	>64	>128	16	0.5	1	0.5	2	0.5	2/1
KP79	>128	>128	>128	32	>64	>128	32	1	1	0.25	1	16	2/1
KP81	>128	>128	>128	128	>64	>128	128	16	32	0.125	4	1	2/1
KP85	>128	>128	>128	32	>64	>128	16	2	1	0.125	1	0.25	2/1
KP89	>128	>128	>128	32	>64	>128	32	1	2	0.125	2	0.25	2/1
KP91	>128	>128	>128	128	>64	>128	64	1	2	0.125	0.5	0.5	1/0.5
KP97	>128	>128	>128	64	>64	>128	128	16	32	0.0625	2	1	2/1
KP9112	>128	>128	>128	64	>64	>128	64	2	2	<0.0625	1	0.5	2/1
KP135	>128	>128	>128	64	64	>128	64	4	2	0.5	2	0.25	2/1
KP8474	>128	>128	>128	>128	>64	>128	64	1	2	0.0625	1	0.5	2/1
KP12339	>128	>128	>128	32	>64	>128	16	1	2	0.125	0.5	0.5	2/1
KP3295	>128	64	>128	>128	>64	>128	>128	32	32	0.25	0.25	0.5	0.25/0.125

*By broth microdilution method for amikacin, aztreonam, fosfomycin, cefoxitin, cefepime, cefotaxime, cefiderocol, levofloxacin, imipenem, meropenem, polymyxin, tigecycline, and ceftazidime/avibactam; iron-depleted cation-adjusted Mueller-Hinton broth in custom-prepared MIC panels (*14*) method for cefiderocol. Breakpoints are according to Clinical and Laboratory Standards Institute 2020 (https://clsi.org) and EUCAST 10.0 (https://www.eucast.org/clinical_breakpoints) guidelines. *Escherichia coli* ATCC 25922 was the quality control strain. AMK, amikacin; ATM, aztreonam; FOF, fosfomycin; FOX, cefoxitin; FEP, cefepime; CTX, cefotaxime; LVX, levofloxacin; IPM, imipenem; MEM, meropenem; CST, colistin; TGC, tigecycline; FDC, cefiderocol; CZA, ceftazidime/avibactam.

### Genomic Features and Plasmid Characterization

**Figure 1:**
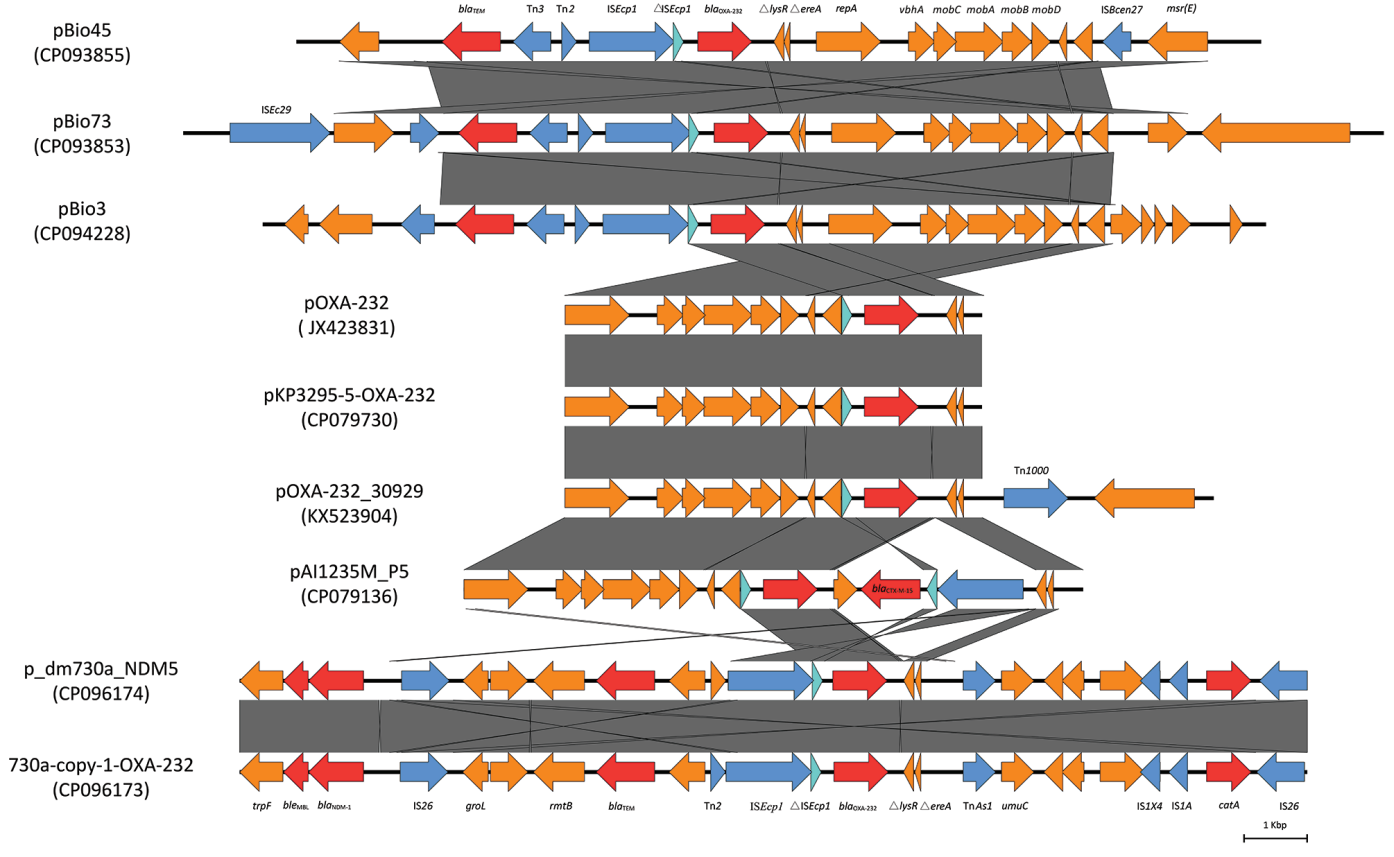
Genetic context of *bla*_OXA-232_ identified in patients in China investigated in study of global phylogeography and genomic epidemiology of *bla*_OXA-232_–carrying carbapenem-resistant *Klebsiella pneumoniae* sequence type 15. Arrows represent coding sequences (red arrows, antimicrobial resistance genes; yellow or blue arrows, mobile elements) and indicate the direction of transcription. Arrow size is proportional to gene length. GenBank accession numbers are provided.

To investigate the genetic characteristics of *bla*_OXA-232_–bearing plasmids, we further subjected isolate KP3295 to whole-genome sequencing using the Oxford Nanopore MinION platform. Whole-genome sequencing data revealed that KP3295 consists of a chromosome of 5,329,783 bp and 10 plasmids (177,848 bp, 133,750 bp, 128,536 bp, 9,730 bp, 6,141 bp, 5,640 bp, 4,510 bp, 3,770 bp, and 3,559 bp). The GC (guanine/cytokine) content of KP3295 was 56.82%; the N50 value was 5,329,783 bp. The *bla*_OXA-232_ gene was carried by a ColKP3-type plasmid (6,141 bp) designated as pKP3295-5-OXA-232. Among the other 20 *bla*_OXA-232_ strains, we identified the *bla*_OXA-232_ gene on ColKP3-type plasmids of comparable sizes (5,934–6,141 bp). The plasmid included 9 open reading frames: *repA*, *mobA*, *mobB*, *mobC*, *mobD*, ΔIS*Ecp1*, *bla*_OXA-232_, Δ*lysR*, and Δ*ereA* (Figure 1). In the conjugation experiment, the *bla*_OXA-232_–carrying plasmid could not be transferred to *E. coli* EC600, which is consistent with the absence of intact conjugal transfer elements in the backbone structure of that plasmid. 

**Figure 2:**
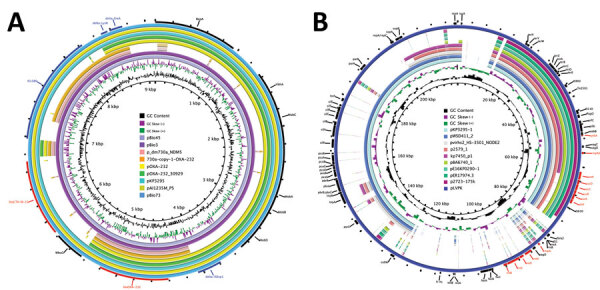
Genetic comparison of *bla*_OXA-232_–carrying plasmids (A) and virulence plasmids (B) recovered from *Klebsiella pneumoniae* isolate KP3295 from China with reference plasmids. A) Alignment of *bla*_OXA-232_–carrying plasmids pKP3295-5-OXA-232 (this study), pAI1235M_P5 (GenBank accession no. CP079136), pOXA-232_30929 (accession no. KX523904), pOXA-232 (accession no. JX423831), pBio45 (accession no. CP093855), pBio73 (accession no. CP093853), pBio3 (accession no. CP094228), p_dm730a_NDM5 (accession no. CP096174), and 730a-copy-1-OXA-232 (accession no. CP096173). We used pAI1235M_P5 as the reference plasmid. Red indicates antimicrobial resistance genes. B) Alignment of virulence plasmids pKP3295-1 (this study), pWSD411_2 (accession no. CP045675), pvirhs2_HS-3501_NODE2 (accession no. OM975892), p2579_1 (accession no. MK649822), kp7450_p1 (accession no. CP090469), pBA6740_1 (accession no. MK649823), pE16KP0290-1 (accession no. CP052259), pER17974.3 (accession no. CP059296), p2723–175k (accession no. CP072940), and pLVPK (accession no. AY378100). We used pLVPK as the reference plasmid. Red indicates virulence genes.

Comparative analysis with the genetic surroundings of the 102 *bla*_OXA-232_–carrying plasmids in the NCBI nucleotide database revealed that the studied plasmid exhibited 100% coverage and 100% identity to plasmid pOXA-232, originally isolated from *E. coli*, which marked the initial discovery of *bla*_OXA-232_. When carried by the transposable elements IS*Ecp1* and Tn*1000*, the compact 6.1 kb *bla*_OXA-232_–carrying plasmid could integrate into larger plasmids, such as pOXA-232_30929 (12,351 bp) reported in the Czech Republic and pAI1235M_P5 (9,117 bp) in India. We found the conserved 6.1 kb ColKP3 plasmid structure in association with the transposable element IS*Ecp1* in IncFIB-ColKP3 (pBio73) and IncFIB-IncHI1B-ColKP3 (pBio45 and pBio3) plasmids from Turkey. Furthermore, with the help of mobile genetic elements, such as IS*Ecp1* and Tn*As1*, the ΔIS*Ecp1*-*bla*_OXA-232_-Δ*lysR*-Δ*ereA* structure was integrated into plasmids from Bangladesh, specifically IncFIB-IncFII (p_dm730a_NDM5 and 730a-copy-1-OXA-232). Those Inc-type plasmids also carried other β-lactam resistance genes, including *bla*_TEM_, *ble*_MBL_, *bla*_NDM-1_, and *bla*_CTM-X-15_ (Figure 1; Figure 2, panel A). 

The 21 CRKP isolates carried several virulence determinants involving yersiniabactin (*fyuA*, *ybtE*, *ybtT*, *ybtU*, *irp1*, *irp2*, *ybtA*, *ybtP*, *ybtQ*, *ybtX*, and *ybtS*), aerobactin (*iutA* and *iucABCD*), and hypermucoidity (*rmpA2*). All 21 isolates belonged to yersiniabactin sequence type 277 (ybST277) and carried *ybt* 16. Except for KP232, KP306, and KP77, all isolates belonged to aerobactin sequence type 1 (AbST1) and contained the aerobactin synthesis locus *iuc* 1. Those virulence determinants were all located on an IncFIB/IncHI1B-type plasmid (177,848 bp), pKP3295-1, in KP3295, which exhibits 99% identity and 91% coverage to classic virulence plasmid pLVPK (Figure 2, panel B). 

### Occurrence and Divergence of ST15 Lineage 

**Figure 3:**
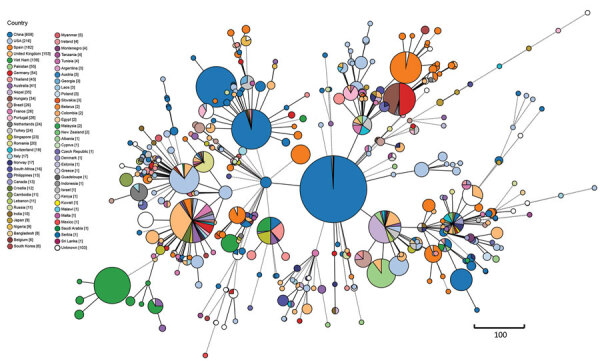
Minimum spanning tree based on core genome MLST analysis of global *bla*_OXA-232_–carrying carbapenem-resistant *Klebsiella pneumoniae* sequence type 15 isolates. Core genome multilocus sequence typing scheme with clade alert distance set as ≤20 alleles. Line lengths connecting each circle depict clonal relationships between isolates. Colors of circles indicate different countries. Numbers in square brackets in the key indicate numbers of isolates recovered from each country. Scale bar represents genetic distance (allelic differences).

We investigated global phylogenetic relationships of 2,118 *K. pneumoniae* ST15 strains using core genome MLST analysis with a cutoff of 20 alleles to define clonality (Appendix 2 Table 1). China, the United States, Ireland, and the United Kingdom emerged as the countries with the highest prevalences of ST15. We identified multiple highly homogeneous strains common between countries, notably United States–China (n = 317), China–Nepal (n = 251), United Kingdom–United States (n = 162), and Vietnam–Thailand (n = 124) (Figure 3). Antimicrobial resistance gene testing identified *bla*_KPC-2_ (19.07%), *bla*_OXA-232_ (15.58%), and *bla*_NDM-1_ (10.57%) as the predominant carbapenem-resistance genes carried by global *K. pneumoniae* ST15 strains. 

We identified all 21 *bla*_OXA-232_ CRKP isolates collected in this study as ST15. We performed phylogenetic analysis between the 21 *bla*_OXA-232_–carrying CRKP isolates and 309 *bla*_OXA-232_–carrying *K. pneumoniae* ST15 isolates obtained from NCBI (Appendix 2 Table 2). The *bla*_OXA-232_–carrying *K. pneumoniae* ST15 isolates originated from 10 different countries: China, United Kingdom, Nepal, Netherlands, Oman, Thailand, United States, France, India, and Australia. Evaluation of clonal relatedness by core-genome SNPs revealed that the *bla*_OXA-232_–carrying *K. pneumoniae* ST15 isolates had an average distance of 29 (range 0–208) SNPs. Phylogenetic analysis using hierarchical Bayesian clustering separated ST15 isolates into 2 clusters, I and II, closely corresponding to China and other countries. Cluster I was further divided into clades 1–4, based on SNP distances ≤20. Among 2 clades from Hangzhou, Zhejiang Province, China, clade 1 had distances of 0–14 (median 4) SNPs and clade 2, 0–20 (median 8) SNPs. Clade 3, primarily from Shanghai, China, had distances of 0–18 (median 10) SNPs. Clade 4, from Hangzhou and Taizhou, Zhejiang Province, showed distances of 0–17 (median 5) SNPs. Strains outside those clades scattered across Shandong Province, Jiangsu Province, Jiaxing in Zhejiang Province, and Shanghai, China, as well as in Nepal. Of note, strains SHK022 and SHK012 from China displayed distances of 13 SNPs, and strains 2703 and 2704 from Nepal, 14 SNPs. Strains from the other 9 countries were predominantly found in cluster II (clade 5), with distances 0–202 (median 100) SNPs. 

**Figure 4:**
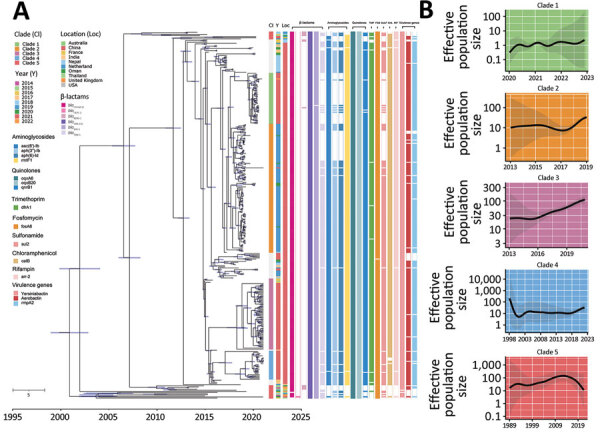
Phylogenetic analysis of global *bla*_OXA-232_–carrying carbapenem-resistant *Klebsiella pneumoniae* sequence type (ST) 15 isolates. A) Time-calibrated maximum clade credibility Bayesian phylogeny based on 330 *bla*_OXA-232_–carrying ST15 recombination-filtered core genomes and distribution of antimicrobial resistance genes and virulence genes in the isolates. The cells with colors indicate presence of the gene; blank cells indicate absence. Different colored circles indicate the geographic location and separation time of strains. Blue bars along branches indicate 95% highest posterior probabilities. B) Effective population size of ST15 lineage strains based on the population structure. Shaded areas indicate 95% highest posterior probabilities. Scale bar indicates number of base substitutions per site.

Subsequently, we analyzed effective population size trajectories for each clade. Clade 1 has steadily ascended since its emergence in 2020. Clade 2 exhibited robust population growth beginning in 2017 and clade 3 in 2016, an increase that projects to clades 2 and 3 dominating global distribution of *bla*_OXA-232_–carrying *K. pneumoniae* ST15 lineage in the future. Clade 4 population sharply declined after emerging, later stabilized, and has increased in recent years. Clade 5 emerged earlier, but its population began to decrease in 2014 (Figure 4). 

In-depth phylogenetic analysis revealed details about origins and mutation rates of *bla*_OXA-232_–carrying *K. pneumoniae* ST15 isolates. The correlation coefficient and R^2^ for the root-to-tip genetic divergence (0.773) compared with time in the TempEst analysis (0.598) indicated a strong linear relationship between accumulated mutations and sampling time, suggesting that enough signal was present to calibrate a strict clock model. The median molecular clock rate was estimated to be 3.40 × 10^−3^ (95% highest posterior density interval [HPD] 3.10–3.69 × 10^−3^) substitutions/site/year, which translates to ≈1.7 mutations/genome/year. The tMRCA for *bla*_OXA-232_–carrying *K. pneumoniae* ST15 lineage was estimated from the temporal height, which dates to 2000 (95% HPD 1996–2003). Strains from China appeared within a recent time window (after 2015) but have greatly increased, apparently the result of a rapidly expanding epidemic of clonal transmission. 

We also compared carriage of antimicrobial resistance genes and virulence genes by *bla*_OXA-232_–carrying *K. pneumoniae* ST15 strains with strains collected globally. Our findings suggest that the *bla*_OXA-232_–carrying *K. pneumoniae* ST15 strains in the study were multidrug-resistant high-risk clones. The ST15 strains carried several genes that confer resistance to β-lactams (*bla*_OXA-232_, *bla*_SHV-106_, *bla*_TEM-1_, and *bla*_CTX-M-15_), aminoglycosides (*aac(6′)-Ib*, *aph(**6**)-Id*, and *aph(3″)-Ib*), chloramphenicol (*catB*), quinolones (*qnrB1*), sulfonamides (*sul2*), 16S rRNA methylase (*rmtF1*), trimethoprim (*dfrA14*), and rifampin (*arr2*). Most strains also exhibited intrinsic antimicrobial resistance, potentially attributed to genes like *fosA6*, *oqxA6*, and *oqxB20*. Of note, strain KP81 collected in this study harbored 2 carbapenemase genes, *bla*_OXA-232_ and *bla*_KPC-2_. In addition, this lineage carries various virulence determinants, including yersiniabactin, aerobactin, and hypermucoidity (*rmpA2*). 

### Transcontinental Dissemination of Epidemic ST15 Lineage in the 21st Century

**Figure 5:**
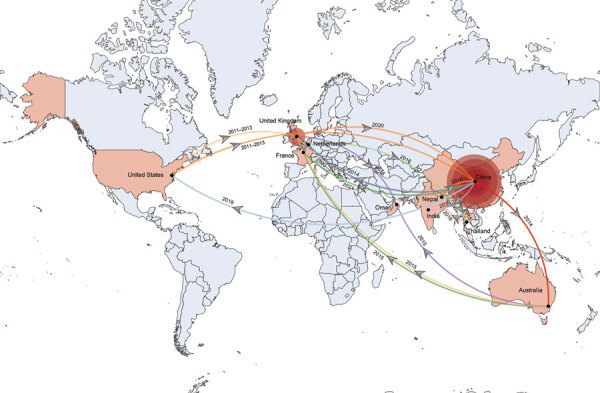
Temporal and spatial transmission trajectory of global *bla*_OXA-232_–carrying carbapenem-resistant *Klebsiella pneumoniae*. Coral-colored countries on map indicate geographic regions where *bla*_OXA-232_ has occurred; arrows show dates and direction of transmission. The *bla*_OXA-232_–carrying isolate originated in the United States, initially expanded to the United Kingdom and China, then spread to the rest of the world, with China as its central focus. Red circles represent major outbreak regions in the United Kingdom and China; size of the red circles corresponds to the number of strains analyzed in each country.

The United States is the likely origin of the ST15 lineage (Figure 5), the offspring of which were introduced into other continents, including Europe, Asia, and Oceania. Transmission from the United States to Europe triggered an epidemic in the United Kingdom. The ST15 lineage was concurrently introduced from the United States to China, where it is the most common strain (93.33%, 308/330 isolates). Epidemic clonal transmissions in China were linked to introductions from the United States (2011–2013), Nepal (2015), and the United Kingdom (2020). Additional global transmission events originated in China and reached the United States, Europe, and Australia. Transmission from China to Australia in 2014 triggered subsequent transmission events from Australia to Europe and Asia. Multiple transmission events occurred from Europe and Australia to Asia in 2015–2016, contributing to complex transmission pathways among countries in Asia. In 2015, strains from Australia reached Oman and subsequently spread to Nepal. In the same year, strains from Nepal spread to Thailand and China. In 2016, strains from Europe were introduced into Thailand and further disseminated to Nepal. After a local outbreak, strains from China spread to India in 2019. 

## Discussion

Worldwide spread of CRKP has been accompanied by considerable incidence and death, posing a severe threat to public health (*29*,*30*). Multiple nosocomial outbreaks of *bla*_OXA-232_–carrying *K. pneumoniae* have occurred in China and may have contributed to the prevalence of those strains (*5*,*6*,*9*,*31*). We analysed the genomic features of 21 CRKP ST15 isolates carrying *bla*_OXA-232_ collected from various regions in China. We integrated the results with global data to investigate the mode of spread and the possible origins of ST15 *bla*_OXA-232_–carrying CRKP. Our findings provide new insights into the genomic characteristics of *bla*_OXA-232_–carrying *K. pneumoniae* and transmission dynamics. 

Our analysis of *bla*_OXA-232_–carrying CRKP genomes found *bla*_OXA-232_ on a 6.1 kb ColKP3 plasmid in isolate KP3295 with high similarity and coverage with previously reported plasmids carrying *bla*_OXA-232_ detected worldwide (*6**,8.13*). Our unsuccessful plasmid conjugation experiment demonstrated the presence of *bla*_OXA-232_ within a nonconjugative plasmid, confirming findings from several previous studies (*7*,*31*). Integration of the *bla*_OXA-232_ gene onto IncF-, IncHI1B-, and IncFII-type plasmids through the transposon element IS*Ecp1* has been documented. IncF- and IncHI1B-type plasmids serve as crucial carriers for multiple antimicrobial resistance genes, such as extended spectrum β-lactamases and *bla*_NDM_, that exhibit the ability for interspecies conjugative transfer. Those plasmids are prevalent among members of Enterobacterales and increase risk for wide dissemination (*32*,*33*). The integration of ColKP3-type miniplasmid on a larger plasmid suggests that, although not self-transmissible, ColKP3 can be mobilized with the assistance of a self-transmissible plasmid. Of note, plasmids encoding *bla*_OXA-232_ are distributed already in South Asia, Europe, and South America. The insertion sequence IS*Ecp1* was completely lost from the plasmid, which may have inactivated IS*Ecp1* transposase and ensured the stability of the *bla*_OXA-232_ gene on the ColKP3 plasmid (*9*). 

Global dissemination of *bla*_OXA-232_ on nonconjugative plasmids, along with escalating outbreaks in China, prompted our investigation into the transmission dynamics and origins of *bla*_OXA-232_–carrying *K. pneumoniae*. A systematic phylogenetic analysis of 2,118 global ST15 strains revealed a high degree of homogeneity among strains originating from different countries, suggesting the dissemination of *K. pneumoniae* ST15 as a globally prevalent clone. Subsequent genomic epidemiologic analysis revealed distinct regional clustering, primarily within China, characterized by minimal SNP distances, suggesting clonal transmission of *bla*_OXA-232_–CRKP ST15 lineage. Clonal transmission may also occur between the strains from China and Nepal based on pairwise distances ≤20 SNPs between these strains. Multiple nosocomial outbreaks of *bla*_OXA-232_–CRKP ST15 across different geographic regions also provided epidemiologic evidence to support our investigation (*5*,*6*,*9*). We also collected 1 *K. pneumoniae* isolate KP81, carrying both *bla*_OXA-232_ and *bla*_KPC-2_, also reported in the United States in 2020 (*34*). Emergence of CRKP ST15 carrying both *bla*_OXA-232_ and *bla*_KPC-2_ in China poses a substantial threat to public health because of its potential for spreading carbapenem resistance internationally through the high-risk clone ST15. *bla*_OXA-232_–carrying *K. pneumoniae* showed low resistance to imipenem and meropenem in our investigation, also described elsewhere (*6*,*9*), possibly attributable to disruption of efficient hydrolytic activity because of loss of a salt bridge between residues D159 and R214 (*35*). 

We used Bayesian phylogeny to elucidate the evolutionary history of *K. pneumoniae* carrying *bla*_OXA-232_ within the ST15 lineage. We estimated that this specific lineage appeared in 2000 (95% HPD interval 1989–1998), approximately the same time the ST147 KL10-O3a (2002) and ST258 (1997) lineages were reported (*36*,*37*). On the basis of our analysis, we suggest that the ST15 lineage originated in the United States. In addition, we successfully reconstructed early transmission events from the United States to both Europe and Asia. Importated strains from the United States and Nepal to China catalyzed clonal spread of strains already circulating in China. From China, strains of ST15 lineage were disseminated across multiple continents and eventually became globally dispersed. Introduction and inappropriate use of fourth-generation cephalosporin (*38*) and carbapenem antimicrobial drugs (*39*,*40*), approved in clinical settings in the 1990s, may have influenced the global emergence and transmission of ST15 lineage. 

Our study was limited by focusing solely on ST15 *bla*_OXA-232_–carrying CRKP strains, which we did because those strains have the highest isolation rate in China and have caused multiple nosocomial outbreaks. Future studies are needed to explore global distribution of other lineages. Second, we might have underestimated prevalence of the *bla*_OXA-232_ gene because of limited carbapenem hydrolysis activity, possibly resulting in false-negative results in antimicrobial susceptibility testing. Finally, although we comprehensively evaluated all publicly available ST15 *bla*_OXA-232_–carrying CRKP strains worldwide, it is possible that some circulating strains went undetected in some regions with dense strain distribution where isolates were derived from a small number of surveillance sites. Therefore, genomic surveillance should be expanded, especially in countries with low isolation rates, to provide a more comprehensive understanding of the emergence, expansion, and spread of CRKP. In conclusion, our research contributes to the comprehension of the global spread of *bla*_OXA-232_–carrying CRKP ST15 lineage and emphasizes the need to develop measures for preventing and controlling progression of the CRKP epidemic. 

Appendix 1 List of global *Klebsiella pneumoniae* strains used in study of carbapenem-resistant *bla*_OXA-232_–carrying *K. pneumoniae* ST15 lineage.

Appendix 2 Lists of global *Klebsiella pneumoniae* ST15 strains and *bla*_OXA-232_–carrying *K. pneumoniae* ST15 isolates used in study of *bla*_OXA-232_–carrying carbapenem-resistant *K. pneumoniae* ST15 lineage.
